# Emerging and Neglected Infectious Diseases: Insights, Advances, and Challenges

**DOI:** 10.1155/2017/5245021

**Published:** 2017-02-13

**Authors:** Nicholas Israel Nii-Trebi

**Affiliations:** Department of Medical Laboratory Sciences, School of Biomedical and Allied Health Sciences, University of Ghana, Accra, Ghana

## Abstract

Infectious diseases are a significant burden on public health and economic stability of societies all over the world. They have for centuries been among the leading causes of death and disability and presented growing challenges to health security and human progress. The threat posed by infectious diseases is further deepened by the continued emergence of new, unrecognized, and old infectious disease epidemics of global impact. Over the past three and half decades at least 30 new infectious agents affecting humans have emerged, most of which are zoonotic and their origins have been shown to correlate significantly with socioeconomic, environmental, and ecological factors. As these factors continue to increase, putting people in increased contact with the disease causing pathogens, there is concern that infectious diseases may continue to present a formidable challenge. Constant awareness and pursuance of effective strategies for controlling infectious diseases and disease emergence thus remain crucial. This review presents current updates on emerging and neglected infectious diseases and highlights the scope, dynamics, and advances in infectious disease management with particular focus on WHO top priority emerging infectious diseases (EIDs) and neglected tropical infectious diseases.

## 1. Introduction

A “disease” is any condition that impairs the normal function of a body organ and/or system, of the psyche, or of the organism as a whole, which is associated with specific signs and symptoms. Factors that lead to organs and/or systems function impairment may be intrinsic or extrinsic. Intrinsic factors arise from within the host and may be due to the genetic features of an organism or any disorder within the host that interferes with normal functional processes of a body organ and/or system. An example is the genetic disease, sickle cell anaemia, characterized by pain leading to organ damage due to defect in haemoglobin of the red blood cell, which occurs as a result of change of a single base, thymine, to adenine in a gene responsible for encoding one of the protein chains of haemoglobin. Extrinsic factors are those that access the host's system when the host contacts an agent from outside. An example is the bite of a mosquito of* Anopheles *species that transmits the* Plasmodium falciparum* parasite, which causes malaria. A disease that occurs through the invasion of a host by a foreign agent whose activities harm or impair the normal functioning of the host's organs and/or systems is referred to as infectious disease [1–3].

Infectious diseases are generally caused by microorganisms. They derive their importance from the type and extent of damage their causative agents inflict on organs and/or systems when they gain entry into a host. Entry into host is mostly by routes such as the mouth, eyes, genital openings, nose, and the skin. Damage to tissues mainly results from the growth and metabolic processes of infectious agents intracellular or within body fluids, with the production and release of toxins or enzymes that interfere with the normal functions of organs and/or systems [4]. These products may be distributed and cause damage in other organs and/or systems or function such that the pathogen consequently invades more organs and/or systems.

Naturally the host's elaborate defence mechanism, immune system, fights infectious agents and eliminates them. Infectious disease results or emerges in instances when the immune system fails to eliminate pathogenic infectious agents. Thus, all infectious diseases emerge at some point in time in a given population and in a given context or environment. By understanding the dynamics of disease and the means of contracting it, methods of fighting, preventing, and controlling are developed [2, 5, 6]. However, some pathogens, after apparent elimination and a period of dormancy, are able to acquire properties that enable them to reinfect their original or new hosts, usually in increasingly alarming proportions.

Understanding how once dominant diseases are reappearing is critical to controlling the damage they cause. The world is constantly faced with challenges from infectious diseases, some of which, though having pandemic potential, either receive less attention or are neglected. There is a need for constant awareness of infectious diseases and advances in control efforts to help engender appropriate public health responses [7, 8].

## 2. Emerging Infectious Diseases

The phenomenon of disease emergence was historically long understood by scientists. This was well expressed by Charles Nicolle, then director of the Institute Pasteur de Tunis, in a talk he gave in 1920 on “Life and Death of Infectious Diseases” to highlight the potential threat that infectious diseases represent [2, 10]. The concept of emerging diseases appeared over time, but began to receive attention in the late 1960s to mid-1970s with the sudden appearance of the viral haemorrhagic fevers such as Crimean-Congo haemorrhagic fever, Lassa fever, and Ebola fever. EID received greater attention with the appearance of other severe syndromes in the 1980s during which unusually big epidemics including HIV/AIDS occurred.

The terms “emerging and reemerging diseases” were formally given by Joshua Lederberg, Robert B. Shope, and Mary Wilson in 1987. The term is used in reference to diseases of infectious origin and whose incidence in humans has either increased within the past two decades or threatens to increase in the near future [11]. A 1992 report by the Institute of Medicine (IOM) on emerging infections, which underscored the microbial threats to health in the United States [1, 2, 12, 13], provided the impetus for current widespread attention on emerging and reemerging infectious diseases. This formal designation seemed to have drawn greater attention and placed the issue of emerging diseases high on the agenda of national and international health programs and has formed a key part of various organizational, institutional, and departmental research focus. The field of emerging disease exploration was strengthened by the creation of “special pathogens branch,” which is a special force on emerging diseases by the WHO at the Centre for Disease Control and Prevention (CDC) in Atlanta, Georgia, USA [14, 15]. The main objective was to spearhead research and related activities aimed at understanding the emergence of new infectious diseases and their reappearance in new populations after a long period of silence and find ways to prevent or control them.

EID thus falls under two major categories—newly emerging and reemerging infectious diseases. Newly emerging infections refer to diseases that have been discovered in the human host or a population for the first time; reemerging infectious diseases can be defined as infectious diseases that reappear, usually in more pathogenic form and in rapidly increasing incidence or new geographic locations after apparent control or eradication [6, 7]. Emerging infections (EIs) have featured prominently in the course of human history; they have caused inestimable harm to humanity [16]. They represent a continued threat to humanity and therefore deserve awareness and preparedness at all times.

## 3. Major Causes of Infectious Disease Emergence

Emergence and reemergence of infectious diseases occur over time. Prior to causing an epidemic, infectious disease agents go through various stages of adaptation to access or acquire pathogenic characteristics in a new host [17]. Specific processes such as gene mutation, genetic recombination, or reassortment as well as factors that compel microbial agents to change reservoir hosts constitute opportunities for infectious agents to evolve, adapt to new hosts in new ecological niches, and spread easily [18, 19]. A number of factors contribute to this adaptation and consequent disease emergence. The complex interactions between infectious agents, hosts, and the environment are key.

Specifically, factors affecting the environment include depletion of forests, expansion and modernization of agricultural practices, and natural disasters such as floods. These potentially lead to changes in microbial ecological niches and fuel microbial adaptation to human host [20, 21]. Sociodemographic factors such as increase in population density, falling living standards, decline of infrastructure, human travel, conflicts and social instability, and killing of wild animals for meat all lead to increase in host-microbe contact, which facilitate infections in humans [22–25]. There are also some pathogens whose emergence is as a result of deliberate human action. These are those employed as biological weapons for destruction and so their emergence is “deliberate.”

Besides host and environmental factors, changes or mutation in the genome of a pathogen, which occurs as a result of exposure to chemicals and antimicrobial agents (e.g., antibiotic), may lead to gene damage [26] and emergence of drug resistant pathogen variants that could cause new disease [18]. Thus, human, microbial, and environmental factors constitute major causes of infectious disease emergence and the virulence or pathogenic potential depends on a complex combination of these factors [27]. However, generally, emerging infectious diseases caused by viral pathogens are responsible for the greatest proportion of the EID threat, having caused about two-thirds of the infectious disease burden and usually characterized by very high epidemics. Examples are Filoviruses, Ebola, and Marburg [28, 29].

## 4. The Emerging Infectious Disease Burden

Infectious diseases (IDs) occupy a prominent position in world history owing to the highly significant burden they present to human survival and development. They constitute a significant proportion of all human diseases known. At least 25% of about 60 million deaths that occur worldwide each year are estimated to be due to infectious diseases [30, 31]. Neglected IDs claim the lives of more than half a million people every year and have rendered at least 1 billion people chronically infected [32]. The scourge of emerging IDs is also well known since ancient times.

There are myriad examples that underscore the gravity of the impact infectious diseases have had on humans. In the Middle Ages the Black Death (1348–1350) killed 30%–60% of Europe's population. In the 18th century, smallpox killed an estimated 400,000 Europeans each year and rapidly decimated and weakened native populations in the Americas and Australia and, in the 20th century, it was responsible for an estimated 300–500 million deaths, prior to its final eradication in the late 1970s [33–35]. Like smallpox, measles has been a scourge for centuries, afflicting millions of people, causing massive destruction to native populations especially in the Americas and Europe over the years. In 1989–91, there were 55,000 cases in a measles outbreak in the US, which led to 11,000 hospitalizations and 123 deaths. In 2000 measles was declared eradicated in the US; however, it continues to circulate in various parts of the world, having caused as many as 114 900 deaths globally in 2014 [36–38]. Even with the availability of a safe and cost-effective vaccine, measles remains one of the leading causes of death among young children. Furthermore, diarrhoeal disease, mainly caused by a variety of bacterial, viral, and parasitic organisms, is the second leading cause of death in children under five years old and is responsible for killing around 760 000 children every year (Ref: WHO, diarrhoeal disease), while, in older children, pneumonia, diarrhoea, and malaria represent the leading causes of death due to infectious disorders.

Influenza is another major infectious disease the world has witnessed. No other infectious disease has ever claimed as many lives as the 1918-1919 Spanish Influenza epidemic, which killed as many as 40 million people worldwide. The 1918-1919 influenza pandemic killed more people than the World War I (the Great War); it killed more people in one year than in four years of the Black Death Bubonic Plague that occurred in 1347–1351 and more people than thirty-five years of the HIV/AIDS pandemic, which caused an estimated 35 million deaths at the end of 2015 [33, 39, 40]. It has been referred to as the most devastating epidemic recorded in world history and a global disaster [41]. Other devastating infectious diseases include haemorrhagic fevers and the 2014 Ebola disease outbreak, which recorded a total 28,638 cases with 11,316 deaths in ten countries worldwide [42–44]. A number of neglected infectious diseases can also be cited, for example, malaria and diarrhoeal diseases, which alone cause about three million deaths in children every year, and tuberculosis, African trypanosomiasis, echinococcosis, schistosomiasis, leishmaniasis, ascariasis, rabies, cysticercosis, and dengue (Table 3). All these cause various forms of disabilities and death especially in developing nations, afflicting more than one billion people and costing developing nations billions of dollars year after year, besides incalculable wounds inflicted on the sufferers [32, 45].

A number of psychological, emotional, and mental effects associate infectious diseases that worsen the plight of people living with an infectious disease [46, 47]. Some infectious diseases such as leprosy bring shame on those affected and make them shunned or maltreated by their communities. Sufferers tend to lose their freedom and worth. Furthermore, loss of capacity to work due to an infectious disease further increases poverty in adults, which consequently may affect children's education [48]. Infectious diseases also affect cognitive development of children, leading to various social vices that ultimately add to the burden created by the disease and thereby consequently worsen poverty. Thus people in developing countries suffer heavily from the burden of ill health and death caused by infectious diseases, infants and children being the most affected [45, 49, 50].

## 5. Transmission

Infectious diseases arise upon contact with an infectious agent. Five major infectious agents have been recognized, namely, bacteria, viruses, fungi, protozoa, and helminths [7, 51]. Various factors can be identified that create opportunities for infectious agents to invade human hosts. These include global urbanization, increase in population density, poverty, social unrest, travel, land clearance, farming, hunting, keeping domestic pets, deforestation, climate change, and other human activities that destroy microbial habitat [22, 52]. For example, in 1987, a large West Africa RVF outbreak in both human and animal populations was attributed to change in the ecological conditions and animal-humans interactions, caused by flooding in the lower Senegal River area due to construction work on the Senegal River [53].

Human engagement in activities that interfere with ecological and environmental conditions continues, thereby increasing the risk of contact with new pathogens. These pathogens are mostly transmitted though intermediate animal hosts such as rodents [1, 54], which gain increased contact with humans as a result of environmental and human behavioural factors (Table 1). Pathogens may be shared through animal urine and droppings which may be aerosolized and infect susceptible vertebrates including humans. Examples are seen in Lassa fever, Hantavirus Pulmonary Syndrome, and the Nipah virus encephalitis (Table 2), whose viral pathogens have been found to coevolve with specific rodent species [55].

The emergence and transmission of an infectious disease pathogen in humans, as exemplified in Table 1, primarily follows a pathway that involves a reservoir host, which may employ a vector or secondary host to contact a native host such as human or animal index case. Specific mechanisms are required for the emergence and transmission process and these mainly include a certain level of host behaviour, pathogen changes, and environmental factors as well as contact and/or spillover between reservoir specimens and the native host [2, 17]. While reservoir and secondary hosts favour transmission to other species including wild animals, domestic animals, and humans, changes in susceptible host behaviour due to population density and biodiversity favour sustained pathogen spread. Epidemics in pandemic proportions emerge due to sustained intersusceptible host or secondary host transmission of pathogens; thus failure to initiate cross-species infection or generate secondary infections interrupts the emergence process [4].

Some infectious agents that have adapted to nonhuman hosts can be transmitted to humans but not from human to human, resulting in what is termed a “dead end” transmission. Thus, most of the important EIs, unlike HIV, are mainly zoonoses, in that they are infections in animals that are transmitted to humans. Others are vector-borne diseases and so require arthropod vector for their transmission from one vertebrate to another. HIV transmission on the other hand is mainly from human to human by heterosexual means. Socioeconomic factors are responsible for bulk of the infections which have been recorded in the developing world [56–58].

In most cases, a combination of risk factors accounts for infectious disease emergence and/or outbreak of epidemic such as the 2014 West Africa Ebola Virus Disease (EVD) outbreak. The EVD outbreak originated at Meliandou, located in Gueckedou District, Guinea (on 26 December 2013 but was not identified as Ebola until 21 March 2014). Meliandou is known as the Forest Region [59, 60] and it is close to an area where the three countries, Guinea, Sierra Leone, and Liberia, share common borders. Much of the surrounding forest area has, however, been destroyed through foreign mining and timber operations. There is freedom of intercountry movement for commercial, social, and cultural activities. There is some evidence suggesting that the destruction of the forest (estimated at more than 80%) brought potentially infected wild animals, including the bat species, which is thought to be the natural reservoir of the virus, into closer contact with human settlements [61, 62]. Epidemiological studies showed that apart from a few cases linked to an original animal reservoir-to-human transmission, all subsequent cases could be traced back to a single transmission chain involving an index case, an 18-month-old boy. With the exception of those involving foreign healthcare workers, most of the cases were relatives or members of a common social cycle. Extreme poverty, a weakened healthcare system and other government institutions as a consequence of years of civil war, lack of basic infrastructure, poor education standards, dysfunctional societal structures, and certain local customs such as washing the body after death before burial [63, 64] all contributed to the emergence of the outbreak and failure to control the epidemic. This exemplifies the complex and interrelated nature of factors involved in infectious disease emergence.

## 6. Current Top Priority Infectious Diseases

As part of a global strategy and preparedness plan (Blueprint) for rapid activation of research and development (R&D) activities during and to prevent epidemics, the WHO convened a meeting of a group of scientists and public health experts in Geneva on 8-9 December, 2015, to prepare a process for prioritization of severe emerging disease pathogens with the greatest risk of epidemic or pandemic potential [65, 66]. The prioritization was based on a number of factors bordering on the likelihood to cause severe outbreaks necessitating public health emergency in the near future and inadequacy or nonavailability of medical countermeasures. Against this background seven diseases prioritized for urgent action are Crimean-Congo haemorrhagic fever, Filovirus diseases (Ebola virus disease and Marburg), highly pathogenic emerging Coronaviruses relevant to humans (MERS Co-V and SARS), Lassa fever, Nipah, Rift Valley Fever, and a “new disease.” The seventh disease, denoted as “a new disease” thus refers to any hitherto unknown disease that may emerge and require urgent action to contain or prevent epidemics. Table 2 gives essential descriptions of the known priority pathogens [67].

Also listed as serious, necessitating action by WHO to help control them as soon as possible, were these three diseases: chikungunya, Severe Fever with Thrombocytopenia Syndrome, and Zika. First discovered in 1947, evidence of vector-borne Zika virus transmission has been reported in 65 countries and territories since 2015; it has thus been declared a health emergency by WHO [68]. Diseases such as HIV/AIDS, tuberculosis, malaria, avian influenza, and dengue, which also have epidemic potential, were not included in the top priority list of pathogens requiring major control and research networks because there are appreciable funding and mechanisms for improved intervention well in place for these disease pathogens. The Flaviviruses, dengue and chikungunya, are however on the WHO current list of neglected tropical diseases (NTDs) [9, 69]. Details about 16 current most neglected tropical diseases are given in Table 3.

## 7. Public Health Response

EIDs and neglected infectious diseases are both driven to a large extent by human, environmental, and ecological factors. NTDs, however, thrive and persist more under conditions of poverty. People affected by neglected tropical diseases are often of low status in terms of public health priorities and lack strong political voice [70, 71]. A reasonable public health response towards addressing infectious disease problem in general therefore aims at addressing the fundamental factors that promote the occurrence and persistence of these diseases, while embarking on appropriate control measures. WHO therefore supports advocacy and awareness and pathogenesis studies and development and deployment of diagnostic tools and therapeutic drugs and vaccines as the pillars of public health response [72–74].

The international community has recognized the need to galvanize investment to enhance preparation and response to infectious disease threats. Recognizing the existence of many tropical, poverty-related diseases, including neglected tropical diseases, affecting the same populations, the 66th World Health Assembly of the WHO in May 2013 adopted a resolution (WHA66.12), which called on Member States to intensify and integrate measures and pursue investments aimed at improving the health and social well-being of affected populations [75]. The adoption by the WHO of a novel research and development plan, the Blueprint, for rapid activation of activities to address future epidemics is another positive step towards preparedness. Furthermore, there are a number of other important initiatives by national and international bodies, organizations, and foundations with vigorous research and financial commitments towards addressing infectious disease threats [76, 77].

Apparently, NTDs were not listed among the United Nation's Millenium Development Goals (MDGs). Fortunately, NTDs were covered through the Bill & Melinda Gates Foundation funding for research and public health projects related to global health [78]. Further initiatives include the founding of the Global Network of various organizations for NTDs in 2006, also with financial support from the Gates Foundation; the adoption of the historic* London Declaration on Neglected Tropical Diseases, *was made, in early 2012, at a meeting of major stakeholders including the World Bank, the Bill & Melinda Gates Foundation, governments, pharmaceutical companies, and other organizations, as well as the WHO commitment to* “*A*ccelerating Work to Overcome the Global Impact of Neglected Tropical Diseases.” *At least US$ 785 million pledge was made by members towards attainment of the goals of the London declaration, which were to accelerate research and development of new drugs for NTDs and to expand effective drug distribution [79, 80]. This is unprecedented.

In terms of disease management, preventive chemotherapy, vector control, and pesticide management and provision of safe drinking water, basic sanitation and hygiene, and education; veterinary public health services are some of the public health strategies employed by the WHO for the control, elimination, and eradication of NTDs [81, 82]. Preventive chemotherapy in developing countries especially is mainly by mass drug administration (MDA) approach. It is the means of combating helminthic infections such as schistosomiasis, ascariasis, Lymphatic filariasis, trichuriasis, onchocerciasis, and trachoma. Periodically, preventive treatment with anthelmintics is given to all at-risk people living in endemic areas in order to reduce the worm burden and hence decrease morbidity and improve the lives of affected populations. This is yielding significant benefits but needs a long-term commitment [83, 84].

The ultimate goal of infectious disease control, however, is to achieve total eradication. With smallpox having been eradicated, and a great wealth of lessons learned from previous epidemic events such as the West African Ebola crisis, there is optimism that eradication is a reality that must be pursued relentlessly.

## 8. Major Advances in Infectious Disease Control Efforts

The progress made over the past century in combating emerging infectious diseases came about as a result of engagement of several disciplines, namely, environmental studies, epidemiology, immunology, public health, social and cultural studies, pharmacology, medicine, molecular biology, chemistry, veterinary science, sociology, and anthropology among others [85–87]. Advances in basic science research and development of molecular technology and diagnostics have enhanced understanding of disease aetiology, pathogenesis, and molecular epidemiology, which provide basis for appropriate detection, prevention, and control measures as well as rational design of vaccine, by which some diseases have been successfully eliminated.

The development of the nucleic acid detection and genome sequencing technology in the nineteenth century has tremendously revolutionized infectious disease research, especially pathogenesis, diagnosis, and treatment and hence optimum patient care and management. A number of molecular assays have been developed for the detection, characterization, and quantitation of the ever-increasing number of infectious pathogens at a faster rate and with higher sensitivity and specificity as compared to traditional methods [2, 88]. From the initial stages of single pathogen detection, nucleic acid amplification methods today have been developed with a high-throughput capacity to generate a wealth of data on various types of pathogens (e.g., bacteria, parasites, and viruses) with specific disease markers (e.g., virulence, antibiotic resistance, and susceptibility factors) present in various types of specimen including blood, stool, swabs, urine, cerebrospinal fluid (CSF) samples, and respiratory secretions. Further, automation of nucleic acid detection technology provides “cutting-edge” platforms, the output of which ultimately greatly impacts patient management [89, 90] and also affords more efficient epidemiological and public health interventions.

Advances in molecular diagnostics and sequencing technology have played pivotal role in the control of many infectious diseases. In HIV disease treatment, for example, measurement of plasma HIV-1 viral load is an important technique for monitoring treatment efficacy [91]; while viral gene sequencing is a crucial method by means of which drug resistance development is monitored in HIV-infected persons on antiretroviral therapy (ART). These techniques have been tremendously instrumental in the current ART success story [92].

The acquisition of genomic and protein data has contributed to successful vaccine design and drug development against most of the infectious disease pathogens. A better understanding of known pathogens and discovery of new or previously unknown infectious diseases has been facilitated through genomic and proteomic studies. Elucidation of the pathogenesis of the malaria parasite* Plasmodium falciparum* and individual's susceptibility or resistance to malaria contributed to the development of malaria vaccine (Mosquirix, the first against a parasitic infection in humans) [93]. Other achievements include the discovery of polio vaccine, anti-HIV drugs, and antimicrobials for various infectious agents like cancer-causing human papilloma virus, meningitis-causing pneumococci, and* Haemophilus influenza *type B; the recent Ebola vaccine represents landmark breakthroughs [12, 94].

Not only has advance in acquisition of genomic data contributed substantially to the development of vaccines and antimicrobials, but also it has important application in deciding and guiding successful treatment. Typical examples can be found in HIV antiretroviral therapy. Assay for the type of coreceptor usage by a patient's predominant virus population, whether CCR5- or CXCR4-tropic virus, is necessary before using the antiretroviral drug Maraviroc, which is a CCR5 coreceptor antagonist [95]; the nucleoside reverse transcriptase inhibitor d, Abacavir (ABC), is associated with drug hypersensitivity reactions. This drug may lead to high rates of myocardial infarction in patients who are positive for human leukocyte antigen (HLA) type B*∗*5701 allele. Safe use of Abacavir therefore requires testing patients genetic data for HLA B*∗*5701 allele [96].

Besides pathogen and human factors, notable milestones have been achieved in the global sociopolitical front in addressing infectious disease problems. Since the dawn of this century concerted efforts have been made globally by global organizations, governments, foundations, and partner bodies towards infectious disease control. The United Nation's decision to “combat HIV/AIDS, malaria, and other related diseases” as part, sixth goal, of the eight MDGs has led to transforming HIV from deadly to chronic, manageable disease. Other global initiatives in the fight against HIV include the United Nations–supported Global Fund to Fight AIDS, Tuberculosis, and Malaria (GFATM), the World Health Organization (WHO) “3 by 5” initiative, and the US President's Emergency Program for AIDS Relief (PEPFAR) [97, 98]. NTDs have also received impressive and unprecedented global attention, with heavy financial and research commitments by major institutions and organizations (see section on public health response) [76, 99].

In real terms, the outcome of advances in response to infectious disease threats reflects in marked progress in infectious disease control and human health protection. The discovery of vaccine about two hundred years ago by Edward Jenner (the English physician) has made it possible to prevent approximately 9 million deaths each year globally through routine immunization [100, 101]. Some vaccine-preventable diseases that are at various levels towards eradication include polio, diphtheria, whooping cough, measles, neonatal tetanus, hepatitis B, and tuberculosis. Others are Rubella, Dracunculiasis (Guinea worm), Lymphatic filariasis (elephantiasis), onchocerciasis (river blindness), and Mumps [37, 51, 102].

WHO has planned to eliminate measles by the year 2020. Polio is currently seen in three countries, Afghanistan, Nigeria, and Pakistan, but efforts are underway for its complete eradication and, down from nearly 3.5 million cases in 1986, today there are just 126 cases of Guinea worm recorded globally [103]; Guinea worm disease could be the second human disease after smallpox to be eradicated.

## 9. The Challenge and the Way Forward

The persistent and unpredictable nature of infectious disease emergence represents a continual challenge. Despite significant advances, especially during the past 2 decades, IDs continue to kill several millions of people each year. New and more virulent pathogens continue to emerge and reemerge. Human, social, political, environmental, technological, microbial, and ecological factors impacting infectious disease upsurge continue to increase [104]. Successful approaches to combating emerging infectious diseases threats require consideration of potential challenges and devise means to address them.

### 9.1. Genetic Variation

Genetic changes in pathogenic microorganisms confer new phenotypic properties that adapt infectious agents to new or old hosts, which may be favoured by changing host and environmental conditions. This enhances infectious disease emergence and reemergence, often causing new pandemics. Influenza viruses are a classic example of emerging and reemerging infectious agents, by their ability to undergo multiple genetic changes and evolve in response to changing host and environmental conditions [2, 51, 65, 105]. The IDs emergence threat thus persists as long as pathogens continue to undergo genetic changes and human and environmental activities that favour pathogen adaptation to infection in humans continue. However, findings suggest that virulence of pathogenic microorganisms may be caused by factors other than genetic variation. In the 2013–2016 West Africa Ebola virus disease outbreak, for example, even though rapid genomic variation could be responsible for virulence and transmission rates, pathogenesis studies did not find significant association between change in the virus with the magnitude of the outbreak, suggesting factors extrinsic to the virus to be responsible [63]. This depicts the complex nature of factors that may lead to infectious disease emergence and the enormity of the infectious disease challenge. There is therefore no promise that advances in infectious disease detection and control strategies can successfully stop new diseases from appearing, as each new disease brings unique challenges. Besides, the fact that so far only one human infectious disease (smallpox) has been successfully eradicated and there are many more whose pathogenesis have yet to be understood suggests that elimination of a disease once it gains way into human population is a hard task that deserves all persistent efforts to terminate its persistence.

### 9.2. Antimicrobial Resistance

Another major problem arising from genetic changes is the development of resistance to drugs [106]. A typical example is seen in HIV. Besides drug-drug interactions and toxic side effects, drug resistance arising from drug pressure coupled with high rate of genomic variation (during viral replication) is a major obstacle in HIV antiretroviral therapy, leading to treatment failure and necessitating regimen switches [107, 108]. Current antiretroviral therapy therefore employs a combination of anti-HIV compounds from at least two classes or drug groups with different mechanisms of action against HIV replication. Combination ART is necessary to suppress plasma HIV viremia, restore immunologic function, and reduce likelihood of drug resistance development for favourable treatment outcomes [109]. The problem of emergence of drug resistant microbes and resistance to antimicrobial agents very well characterizes many bacterial infectious agents such as* Escherichia coli*,* Pneumococcus*,* Neisseria gonorrhoeae*, and* Staphylococcus aureus*. Many well known antibiotics no longer clear bacterial infections due to microbial resistance. Evolution of drug resistant pathogens thus necessitates continued development of new antiviral and antimicrobial products. As such for HIV alone there are currently at least 25 anti-HIV compounds licensed for the treatment of AIDS [110].

### 9.3. Surveillance

This nature of infectious disease challenge calls for constant surveillance and timely intervention. There is need to develop and effectively deploy vaccines and drugs where they are needed; there is also the need for necessary infrastructure and skilled personnel to support prompt diagnosis and a need for ongoing research to aid development of effective countermeasures. However, given the extensive distribution of pathogens [15, 67, 111], some of which are not yet known or fully described, and the variety of animal species involved, effective surveillance and control of IDs constitute a significant public health challenge, and also predicting zoonotic emerging disease events remains a subject requiring persistent scientific exploration.

### 9.4. The Way Forward

There are a number of lessons to be learned from past epidemics to help our appreciation of the unpredictable and devastating nature of IDs. Infectious disease-causing pathogens have demonstrated sufficiently their capacity to emerge and spread rapidly by any possible means across borders, exhibit high pathogenic potential, and evolve or mutate to resist drug attack. This calls for efficient armament at any time. This can be achieved through greater international cooperation; effective local, regional, and global networks for strong infectious disease surveillance and research collaboration to enable sharing of biological and study materials to enhance antimicrobial product development and vaccine trials; collaboration between animal and human health sciences to strengthen capacity for identification of microbial agents with epidemic potential so as to prevent their emergence; stable society; committed medical and political leadership; and resource prioritization. There is also the need to focus special attention on situations that promote disease emergence, especially human activities, that degrade environmental and alter ecological conditions, which increase animals contact with humans. These are vital for a meaningful pandemic preparedness.

## 10. Conclusion

Emerging and neglected infectious diseases are a real public health threat, and infectious disease outbreaks can have serious social, political, and economic effects. Much have been learned from previous outbreak events and far-reaching advances have been made since the landmark IOM report [11], which underscored the important concept of emerging infectious diseases. Pandemic preparedness however remains a major global challenge. A complex number of factors relating to human behaviour and activities, pathogen evolution, poverty, and changes in the environment as well as dynamic human interactions with animals have been found to contribute to infectious disease emergence and transmission. Aggressive research is warranted to unravel important characteristics of pathogens necessary for diagnostics, therapeutics, and vaccine development and possibly enable detection of those pathogens with the potential to cause epidemic. National and international organizations networking, effective interagency and international research collaborations, appropriate financial support of the public health infrastructure, and poverty reduction are very vital for addressing emerging and neglected infectious disease threats.

## Figures and Tables

**Table 1:** Some past emerging infectious disease epidemics and probable factors for outbreak.

Year	Emerging disease	Pathogenic agent	Main probable factor
1958	Argentine haemorrhagic fever	*ArenavirusJunin* virus	Changes in agricultural practices of corn harvest (maize mechanization)

1981	Acquired immunodeficiency syndrome (AIDS)	Human immunodeficiency virus	Sexual contact/exposure to blood or tissues of an infected person

1959	Bolivian haemorrhagic fever (BHF)	*ArenavirusMachupo* virus	Population increase of rats gathering food

1983	Crimean-Congo haemorrhagic fever	CCHF virus	Ecological changes favouring increased human exposure to ticks of sheep and small wild animals

1996	Haemorrhagic colitis	*Escherichia coli *O157:H7	Ingestion of contaminated food, undercooked beef, and raw milk

1976	Malaria	*Plasmodium falciparum*	Human behaviour/rainfall and drainage problems/mosquito breeding/neglect of eradication policy, economics, and growing interchange of populations

1993	Hantavirus pulmonary syndrome (HPS)	*HantavirusSin Nombre virus*	Human invasion of virus ecological niche; close contact with infected rodent natural reservoir; inhalation of infectious aerosolized rodent faces and urine

1997	Highly pathogenic avian influenza (HPAI)	H5N1 virus	Animal-animal influenza virus gene reassortment; emergence of H5N1 avian influenza, extensive chicken farming

1889, 1890, 1918, 1957	Pandemic Influenza	*Paramyxovirus* influenza A	Animal-human virus reassortment and antigenic shift

1969	Lassa fever	*Arenavirus* Lassa virus	Hospital exposure to index case—rodent exposure

1956	Marburg disease	*Filovirus *Marburg virus	Trade (and use of wild imported monkeys); use of animal organs for specific purpose

2003	Severe acute respiratory syndrome (SARS)	SARS *Coronavirus*	Hunting and feeding on infected wild animals (viverrids)

1987	Rift Valley fever (RVF)	*Bunyavirus* RVF virus	Dramatic increase in mosquito vector breeding sites (by dam filling); weather (rainfall) and cattle migration (guided by artificial water holes)

1976	Ebola haemorrhagic fever	*Filovirus* Ebola virus	Rainforest penetration by humans/close contact with infected game (hunting) or with host reservoirs (bats)/infected biological products/nosocomial/needle spread

1953	Dengue haemorrhagic fever (DHF)	Dengue viruses 1, 2, 3, and 4	Increasing human population density in cities in a way that favours vector breeding sites (water storage), for example, *Aedes aegypti*

*Note*: adapted from “Encyclopedia of Infectious Diseases-Modern Methodologies” [2].

**Table 2:** Top priority emerging infectious diseases.

Emerging disease	Crimean-Congo haemorrhagic fever	Ebola virus disease & Marburg haemorrhagic fever	Middle East Respiratory Syndrome & SARS	Lassa Fever	Nipah	Rift Valley Fever
Year of (re)emergence	(1) 12th Century, (2) 1944-45 & 1967	Ebola: 1976 Marburg: 1967	MERS: 2012 SARS: 2002	Isolated in 1969	1998-1999	In livestock: 1910s In humans: 1931 (all in Kenya)

Causative organism	*Nairovirus (CCHF virus)*	EVD: Ebola virus MHF: Marburg virus	MERS: MERS-CoV	Lassa virus	Nipah virus (NiV)	Rift Valley Fever (RVF) virus

Type/class of organism	Virus (*Bunyaviridae*)	Virus (*Filoviridae*)	Virus (Coronaviridae)	Virus (Arenaviridae)	Virus (Paramyxoviridae)	Virus (Bunyaviridae)

Vector/animal host	*Hyalomma* tick, Domestic animals	Fruit bats species EVD: Pteropodidae MHF: *Rousettus aegypti*	Not well known; camel is implicated as reservoir host	*Mastomys* rats	Fruit bats of genus *Pteropus*	(1) Mosquito species, mainly *Aedes* sp. (2) Domestic remnant

Epidemiology	Fatality rate of 10–40%, at least 140 outbreaks & >5000 cases since 1967	EVD: about 50% fatality. At least 31,076 cases with 12,922 deaths since 1976 MHF: case fatality rate is up to 88%	MERS: case fatality approx. 36%; severe in people with weakened immune systems, with chronic diseases	At least 100,000 cases annually in the endemic regions of West Africa with case fatality rates of 5–10%	At least 477 people infected, 252 killed since 1998; case fatality rate of 40–70%	Commonly affects livestock, causing disease, abortion, and death in thousands of domesticated animals

Populations at risk	Endemic in Africa, the Balkans, the Middle East, and Asia	EBV: Africa MHF: Frankfurt in Germany; Belgrade and Africa	MERS: people with chronic disease. Countries include Egypt, Oman, Qatar, and Saudi Arabia	Endemic in West Africa	Southeast Asia region	Africa and Arabian Peninsula

Mode of transmission	(1) Tick bites (2) Zoonotic: contact with infected animal blood and with secretions or body fluids of infected persons	EBV: contact with blood, secretions, and body fluids/organs of infected (a) nonhuman primates and (b) humans MHF: mainly human-to-human	MERS is zoonotic: no human-to-human transmission; origin and exact route are unknown	Zoonotic: (1) exposure to urine or faeces of infected *Mastomys *rats (2) Direct contact with body fluids of infected person	(1) Contact with excretion and secretion of infected bats (2) Direct contact with infected pigs	Zoonotic and Epizootic: Bite of infected mosquitoes (in humans and animals)

Clinical presentations	Nonspecific: high fever, myalgia, headache, nausea, abdominal pain, and nonbloody diarrhoea	Clinically similar: fever, severe headache, diarrhoea, lethargy, and so on; impaired kidney function, internal /external bleeding, and nervous system problems (MHF)	Ranges from no symptoms to death. Generally fever, cough, and shortness of breath; pneumonia, gastrointestinal problems, and respiratory failure	About 80% of infections are asymptomatic. Symptoms are variable: fever, cough, malaise; pains, fluid in the lung cavity, facial swelling, bleeding, and more	(1) Barking pig syndrome (in pigs) (2) In humans: fever, muscle pain; brain inflammation leading to coma	Humans: include fever, muscle/joint pain; eye disease, meningoencephalitis, and haemorrhagic fever Animals: mortality and abortion

Pathogenesis	Not well understood	Not well understood	Not well understood	Not well understood	Not well understood	Not well understood

Diagnosis	(1) Virus isolation by cell culture (2) Viral genome detection (RT-PCR) (3) Serology (ELISA)	(1) Virus isolation by cell culture (2) Viral genome detection (RT-PCR) (3) Serology (e.g., ELISA)	(1) Mainly by molecular detection (RT-PCR) (2) Also serology (ELISA)	Only in reference labs: isolation by cell culture, viral genome detection (RT-PCR), and serology (ELISA & neutralization)	(1) Virus isolation (2) Histopathology (3) RT-PCR (4) Serology (ELISA, neutralization)	(1) Isolation by cell culture (2) Viral genome detection (RT-PCR) (3) Serology (ELISA)

Treatment	No vaccine available: (1) General supportive care management (2) Ribavirin antiviral	No vaccine or specific antiviral treatment. General supportive care management	MERS: no vaccine or specific treatment. General supportive care management	No vaccine yet (1) Early supportive care (2) Ribavirin antiviral therapy seems effective	No vaccine available (1) Symptoms management; (2) Ribavirin antiviral treatment	No specific treatment; generally supportive therapy

Prevention	Minimize tick burden in livestock, minimize human contact with vertebrate hosts	Awareness EVD: good hygiene; avoid contact with blood and body fluids MHF: protect pigs from fruit bats contact	MERS: general hygiene measures in contacting camels/other animals; avoid consumption of raw or undercooked animal products	(1) Good hygiene—in community, homes (2) Standard infection prevention and control measures in healthcare and lab settings	Avoid contact with infected secretions, excretions, blood, or tissues of infected pigs and bats	(1) Animal vaccination (2) Protection against vector bites (3) Animal health surveillance

*Note*. Source of list: http://www.who.int/medicines/ebola-treatment/WHO-list-of-top-emerging-diseases/en/ [9].

MERS: Middle East Respiratory Syndrome; SARS: Severe Acute Respiratory Syndrome.

**Table 3:** Major neglected tropical diseases.

Disease	Causative agent	Transmission	Pathogenesis	Affected populations	Treatment and management	Prevention/public health response
Buruli ulcer	*Mycobacterium ulcerans *(Bacterium)	Mode unknown	Destruction of skin & soft tissues, leading to ulcer	Poor rural communities; more in Africa, 33 countries	Rifampicin, streptomycin/amikacin, or surgery	Early detection and antibiotic treatment

Chagas disease	*Trypanosoma cruzi* (protozoan parasite)	Vector-borne	Cardiac or mixed alterations in chronic infection	~8 M people infected worldwide, mostly Latin America	Curable with benznidazole and nifurtimox	Vector (triatomine bug) control

Dengue and chikungunya	Dengue virus sp.: DENV 1, DENV 2, DENV 3 & DENV 4 (Flaviviruses)	Dengue & chikungunya: mosquito sp. (*Aedes aegypti & Ae. albopictus) *	Fatal: plasma leaking, bleeding, and organ impairment	Worldwide, ~100 countries; 50–100 mil infections/year. Esp. Asia & Latin America	Dengue: vaccine available; chikungunya: no vaccine	Control of mosquito vectors

Dracunculiasis (Guinea worm disease)	*Dracunculus medinensis* (Nematode parasite)	Drinking water containing parasite-infected water-fleas (*Cyclops*)	Rarely fatal Leads to oedema and ulcer, usually of the feet	22 cases in 4 African countries in 2015	No vaccine available yet	Improved drinking water sources. Near eradication

Echinococcosis	*E. granulosus *and *E. multilocularis *(*Echinococcus *parasites)	Through faeces of dogs, foxes & other carnivores	Involves liver and other organs. Progressive and fatal if untreated	>I M people worldwide affected at any one time	Expensive and complicated to treat	Complex Regular deworming of domestic carnivores helps

Endemic treponematoses (Yaws)	*T. Pallidum* subspecies *pertenue *(bacterium)	Person-to-person (nonsexual) with infected fluid	Disfigurement of the nose and bones; hyperkeratosis	Africa, Asia, Latin America, and the Pacific	Azithromycin Benzathine penicillin	No vaccine: early diagnosis and targeted treatment

Foodborne trematodiases	Trematode parasite sp*. Clonorchis*, *Opisthorchis*, *Fasciola,* and *Paragonimus*	Zoonotic: consumption of raw/poorly cooked food	Organ-specific, reflects adult worm final location	>70 countries worldwide, mainly East Asia and South America	Use of anthelminthic medicines	Reduce infection risk; control associated morbidity

Human African trypanosomiasis (sleeping sickness)	Trypanosoma parasite sp. *T. brucei gambiense *(98%) *T. brucei rhodesiense* (2%)	By bites of infected tsetse fly (of Glossina genus)	Affects central nervous system, causing neurological (and sleep) disorders	Occurs in 36 sub-Saharan Africa countries (>70% of cases occur in DR Congo)	Drugs available. Depends on disease stage and parasite species	Free antitrypanosome medicines provided by WHO; efforts at elimination

Leishmaniasis	Protozoan *Leishmania* parasites (over 20 species)	Bite of infected female phlebotomine sandflies	Infection rarely leads to disease development	Worldwide. Risk increased by poverty	Complex; depends on several factors	Complex; combination of intervention strategies

Leprosy (Hansen disease)	*Mycobacterium leprae* (bacterium)	By air (from nose & mouth) through close contact	Damage of peripheral nerves leading to paralysis	Southeast Asia Region	Multidrug (Dapsone, rifampicin & clofazimine)	Early diagnosis and treatment to avert disability

Lymphatic filariasis (elephantiasis)	* Filarioidea *nematodes: *Wuchereria bancrofti*, *Brugia malayi*, and *B. timori*	By mosquito sp. (*Culex*, *Anopheles*, and *Aedes*)	Invade lymphatic system; disrupt immune system	WHO Southeast Asia & Africa; >120 M people	Albendazole + ivermectin/diethylcarbamazine citrate	Morbidity management; mass drug administration

Onchocerciasis (river blindness)	*Onchocerca volvulus *(parasite)	Bite of blackfly (*Simulium damnosum* sp. mainly)	Parasites migrate throughout the body, casing a variety of symptoms	36 countries: Africa, Arabian peninsula & Americas	Ivermectin (manufactured Merck & Co, free)	Yearly ivermectin administration to affected populations

Rabies	Rabies virus	Bites/scratches of affected domestic/wild animals	Affects central nervous system, leads to death	All continents but Antarctica. Mostly in Asia & Africa	Postexposure prophylaxis (with vaccine course)	Preventive immunization of people; vaccinating dogs

Schistosomiasis (Bilharzia)	*Schistosoma* parasite sp.: *S. haematobium, S. mansoni, and S. japonicum*	Contact with infested fresh water bodies	Live in blood vessels, body tissues & damage organs	At least 90% of estimated cases are in Africa	Drug available: Praziquantel therapy	Good water/sanitation, snail control; preventive therapy

Soil-transmitted helminthiases	*Helminth *parasite* species Ascaris lumbricoides, Trichuris trichiura, Necator americanus & A. duodenale*	Parasite eggs in human faeces-contaminated soil	Intestinal damage and blood loss. Rarely fatal	Worldwide: esp. sub-Saharan Africa, the Americas, China, and East Asia. ~2 billion people	Medicines available: Albendazole and Mebendazole	Deworming people at risk, improved sanitation, education

Taeniasis/ cysticercosis	*Taenia* (tapeworm) parasites *T. solium (pork tapeworm)T. saginata* (beef tapeworm)	Ingestion of larval cysts through contaminated food (pork or beef) or water	Intestinal; central nervous system attack. Can be fatal	Africa, Asia, and Latin America	Drug available: Praziquantel and niclosamide	Veterinary, human health, and environmental approach

Trachoma	*Chlamydia trachomatis* (bacterium)	Eye-seeking flies, poor water and sanitation	Leading infectious cause of blindness globally	51 countries, 1.2 M people blind, 232 M at risk	Surgery, antibiotics, and facial cleanliness	Environmental improvement; target: elimination by 2020

*Note*. M: million.

Source of list: http://www.who.int/neglected_diseases/diseases/en/.
